# Does *Hukou* origin affect establishment of health records in migrant inflow communities? A nation-wide empirical study in China

**DOI:** 10.1186/s12913-018-3519-6

**Published:** 2018-09-10

**Authors:** Yangyang Qian, Dandan Ge, Li Zhang, Long Sun, Jiajia Li, Chengchao Zhou

**Affiliations:** 1 School of Public Health, Shandong University, Jinan, 250012 China; 2 School of Public Health, Key Lab of Health Economics and Policy Research, Shandong University, 44 Wen-hua-xi Road, Jinan, 250012 Shandong China

**Keywords:** *Hukou*, Health records, Internal migrants, Determinants

## Abstract

**Background:**

With the implementation of Chinese economic reform and rapid urbanization, policies and values surrounding migration have changed and given rise to unprecedented population mobility. This study is designed to examine the effect of *Hukou* origin on establishment of health records among internal migrants in China.

**Methods:**

The data used for this study are from the 2015 National Internal Migrant Population Dynamic Monitoring Survey, covering 112,782 migrants nationwide. For continuous variables, the *p* value is calculated using Student t test; for categorical variables, the p value is calculated using chi-square test. Binary logistic regression with an enter method is employed to assess the association of establishment of health records with origin residence.

**Results:**

About 35.1% of the migrant population has established health records in their inflow communities, with 37.4% established among those of urban origin and 34.8% established among those of rural origin. The establishment of health records is significantly higher among migrants of urban origin than among migrants of rural origin (OR = 1.057; 1.017–1.098). Our results also show that among populations of both rural and urban origin, inter-province migrants, along with migrants who are employers, have no plans for long-term residence, have no insurance, and have more family income less likely to establish health records.

**Conclusions:**

This study demonstrates that residence is associated with establishment of health records among the migrant population in China. Targeted policies should be made to improve the establishment of health records among migrants of both rural and urban origins.

**Electronic supplementary material:**

The online version of this article (10.1186/s12913-018-3519-6) contains supplementary material, which is available to authorized users.

## Background

Before the 1980s, migration was not very common within China due to institutional restrictions, undeveloped transportation, and cultural traditions of attachment to one’s native land [1]. However, with the implementation of Chinese economic reform and rapid urbanization, policies and values surrounding migration have changed and given rise to unprecedented population mobility. It is estimated that the internal migrant population reached 245 million in China at the end of 2016 [2]. However, compared with permanent residents, migrants are worse off in terms of accessibility to public health services. Migrant populations have poorer maternal health care, higher rates of infectious diseases, and poorer management of childhood immunization [3]. In 2014, the Chinese central government issued a regulation which indicated that 11 basic public health services should be fully and freely provided to migrants to improve health care utilization, including the establishment of health records [4]. The responsibility of establishing health records for the migrant population has largely fallen to community health centers. They can help health providers and patients to make appropriate decisions for health management or treatment. Recently, health records have also been used to facilitate the management of chronic diseases. It is a constituent part of community health service [5].

Health records are important health information management tools that enable individuals to store, retrieve, and manage their personal health information and ultimately, to make decisions related to their health [6, 7]. Health records include basic personal information, health examination information, health management records, and other records of medical and health services [8]. The primary beneficiaries of health records are individuals with chronic diseases, and those who are elderly and disabled [9]. Health records are helpful for supporting, prevention, and early detection of chronic diseases. Patients can use health records to improve their health and effectively manage their diseases. In addition, health records can also help caregivers to maintain an ongoing connection with patients in order to offer more customized health services.

*Hukou* is the household registration system in China, designed in the 1950s to restrict internal population migration, and especially rural-to-urban migration. A *Hukou* record officially identifies a person as a resident of a particular place. Residents of China are mainly categorized into two types, rural *Hukou* (also called agricultural *Hukou*) and urban *Hukou* (also called non-agricultural *Hukou*). The *Hukou* system is an important institution for regulating population migration. Since the 1980s, more and more Chinese citizens live and work illegally outside their officially registered areas (called “non-*Hukou* migrants”) and as a result have far less eligibility to public services. In the 1990s, a reform started to relax the system to allow some residents to legally migrate to new places with the official authorization of a temporary residency permit. Thus, in the past two decades a new subgroup, *Hukou* migrants, has emerged in China. Even though *Hukou* migrants are authorized by the government to live or work legally within a new place, they face similar barriers to non-*Hukou* migrant when it comes to accessing public services. Their eligibility for most welfare benefits (e.g., health care, education, labor, housing, and social subsidies) remains poorer in local communities than that of permanent residents. Thus, some researchers have suggested that internal migrants should be distinguished by their origins of place, not their authorization status. That is to say, *Hukou* origin may be an important status marker among migrants that deserves more attention by scholars.

Many previous studies have explored factors associated with use intentions of personal health records. Factors associated with use intentions of personal health records included patient satisfaction with the provider, interactions between environmental factors, and personal tendency [10, 11]. Researchers also have identified factors associated with adoption of health records from the perspective of both health care providers and patients. From caregivers’ perspective, factors associated with adoption of health records included practicing in a medically underserved location, geographic health professional shortage areas, being an international medical graduate, privacy concerns, and social influence [12, 13]. From patients’ perspective, influencing factors related to establishment of health records were counties and townships of residence, satisfaction about health education, age, education, housing status, monthly income, and self-reported health [14, 15]. However, most of the existing studies only focus on health care providers or the general population. To date, few studies have focused the establishment of health records among internal migrants in China, and no studies have explored the disparity in establishing health records between migrants with different *Hukou* origin.

To fill this gap, our study aims to explore the effect of *Hukou* origin on the establishment of health records for internal migrants in China. To do so, we have the following specific objectives. First, we will explore the establishment of health records in inflow communities among the migrant population of China. Second, we will look for differences in the establishment of health records between migrants with different *Hukou* origin. Third, we will identify the factors associated with establishment of health records among migrants of rural and urban origin respectively.

## Methods

### Data

The data used for this study are from the 2015 National Internal Migrant Population Dynamic Monitoring Survey (NIMPDMS), conducted by the National Health and Family Planning Commission of China. The overall goal of the survey was to understand accessibility for migrants of public health and family planning services. It was a cross-sectional, nationally representative survey of internal migrants aged 15+ who had been living in the sample cities for more than one month in 2015. The study was conducted in 31 provinces, municipalities, and autonomous regions, and migrant gathering areas in Xinjiang Production and Construction Corps.

The participants of this study were selected by using a stratified multi-stage sampling method with a probability-proportional-to-size (PPS) approach [16]. Face-to-face interviews were conducted by the interviewers who were trained by local health bureau staff. In total, 112,782 migrants with rural or urban origin were included in the analysis.

### Variables and measures

#### Dependent variable

The dependent variable is establishment of health records, which is evaluated based on interviewees’ answers to ‘Do you have established health records in your local community?’ If the response was ‘no, I have never heard of this’ or ‘no, but I have heard of this’, the establishment of health records was coded as ‘no.’ On the contrary, if the answer was ‘I have established health records, the establishment of health records was categorized as ‘yes.’

#### Independent variables

*Hukou* origin is distinguished by migrants’ original place of residence. Participants from urban areas were coded as “urban origin”, and participants from rural areas were coded as “rural origin.”

Demographic characteristics were also used, including age, gender (male vs. female), marital status (single vs. married), and ethnic group (Han vs. ethnic minority).

Socio-economic characteristics used include education (middle school or below vs. high school or above), number of children (< 2 vs. ≥2), employment status (employee, employer, self-supporting laborers, and others), and household income (Q1, Q2, Q3 and Q4, Quartile 1 (Q1) is the poorest and Quartile 4 (Q4) is the richest).

Migration characteristics used include mobility area (inter-provincial vs. intra-provincial), length of migration (≤1 year vs. > 1 years), and plans for long-term residence of more than 5 years (yes, no, and not decided yet).

### Statistical analysis

Statistical analyses were performed using SPSS 21.0. For continuous variables, the *p* value was calculated using Student’s t test; for categorical variables, the p value was calculated using the chi-square test. Binary logistic regression with an enter method was employed to assess the association of establishment of health records with residence. All reported CIs were calculated at the 95% level. Statistical significance was set at the 5% level. Sampling weights were included in all analyses to adjust for the complex survey design.

### Limitations

There are several limitations to this paper. First, information about the establishment of health records and household income was self-reported, thus leading to the possibility of subjective bias. Second, although we are able to identify the association between *Hukou* origin and establishment of health records in migrants, we are unable to identify the exact cause of the observed association. Third, the study sample is specific to China, thus the results from this study are not necessarily generalizable to other contexts.

## Results

Table 1 shows basic information about the 112,782 migrants in our sample. About 35.1% of the migrant population have established health records in their local communities, and the percentage is 37.4% among those of urban origin and 34.8% among those of rural origin. The average age of the migrant population is 37.23, with a standard deviation of 8.38. Generally speaking, the majority the migrants in our sample population are male (58.6%), Han (93.1%), with an education level of middle school or below (69.3%), married (97.2%), are inter-provincial migrants (51.2%), have lived for over 1 year in their current residence (74.3%), have plans for long-term residence (63.2%), have less than 2 children (59.5%), have work status of “employee” (50.7%), have insurance (94.0%), and are in Quartile 2 of the income distribution (35.8%).

**Table 1:** Socio-demographic characteristics of migrant population in China (*n* = 112,782)

Characteristics	Total n (%)	Rural n (%)	Urban n (%)	*χ*^*2*^/t	*P*
N	112,782(100.0)	99,650(85.7)	16,132(14.3)		
Health records				41.689	**0.000**
Yes	39,637(35.1)	33,605(34.8)	6032(37.4)		
No	73,145(64.9)	63,045(65.2)	10,100(62.6)		
Age	37.23(8.38)	37.18(8.42)	37.51(8.12)	6.458	**0.000**
Gender				1.412	0.237
Male	66,110(58.6)	56,585(58.5)	9525(59.0)		
Female	46,672(41.4)	40,065(41.5)	6607(41.0)		
Ethnic group				31.395	**0.000**
Han	104,986(93.1)	89,802(92.9)	15,184(94.1)		
Ethnic minority	7796(6.9)	6848(7.1)	948(5.9)		
Education				13,781.375	**0.000**
Middle school or below	78,210(69.3)	73,387(75.9)	4823(29.9)		
High school or above	34,572(30.7)	23,263(24.1)	11,309(70.1)		
Marital status				246.362	**0.000**
Single ^a^	3115(2.8)	2367(2.4)	748(4.6)		
Married ^b^	109,667(97.2)	94,283(97.6)	15,384(95.4)		
Movement area				58.584	**0.000**
Inter-provincial	57,768(51.2)	49,875(51.6)	7893(48.9)		
Intra-provincial	55,014(48.8)	49,775(48.4)	8239(51.1)		
Time in inflow area				14.918	**0.000**
≤ 1	29,038(25.7)	25,308(26.2)	3730(23.1)		
> 1	83,744(74.3)	71,342(73.8)	12,402(76.9)		
Plans for long-term residence (> 5 years)				524.115	**0.000**
Yes	71,237(63.2)	59,758(61.8)	11,479(71.2)		
No	12,748(11.3)	11,232(11.6)	1516(9.4)		
Not decided yet	28,797(25.5)	25,660(26.5)	3137(19.4)		
Number of children				4124.906	**0.000**
< 2	67,063(59.5)	53,763(55.6)	13,300(82.4)		
≥2	45,719(40.5)	42,887(44.4)	2832(17.6)		
Employment status				1590.730	**0.000**
Employee	57,142(50.7)	47,098(48.7)	10,044(62.3)		
Employer	9850(8.7)	8207(8.5)	1643(10.2)		
Self-supporting laborers	44,046(39.1)	40,000(41.4)	4046(25.1)		
Others	1744(1.5)	1345(1.4)	399(2.5)		
Health insurance				428.804	**0.000**
Yes	106,065(94.0)	91,470(94.6)	14,595(90.5)		
No	6717(6.0)	5180(5.4)	1537(9.5)		
Family monthly income in the last year ^c^				1874.309	**0.000**
Q1	28,485(25.3)	25,439(26.3)	3046(18.9)		
Q2	40,403(35.8)	35,524(36.8)	4879(30.2)		
Q3	22,641(20.1)	19,389(20.1)	3252(20.2)		
Q4	21,253(18.8)	16,298(16.9)	4955(30.7)		

Age is presented as Mean(Standare deviation); The *P*-values in boldface mean statistical significance

^a^Single includes those who are divorced (2.3%) and widowed (0.5%)

^b^Married includes those who are first married (95.5%) and remarried (1.7%)

^c^Quartile 1 (Q1) is the poorest and Quartile 4 (Q4) is the richest

We present our results in two models so that we can better understand the effect of *Hukou* origin on establishment of health records among migrants in China (see Table 2). Model 1 shows that establishment of health records is significantly higher in migrants with urban origin than in migrants with rural origin (OR = 1.120; 95CI 1.082–1.160). Even after controlling for all other variables, establishment of health records is still significantly higher in migrants with urban origin than in migrants with rural origin (OR = 1.057; 1.017–1.098).

**Table 2:** Association of residence and health records in migrant population in China

Characteristics	Model 1 (No covariates)		Model 2 (Covariates)	
	OR (95%CI)	*P*	OR (95%CI)	*P*
Hukou				
Rural	1.0		1.0	
Urban	1.120(1.082–1.160)	**0.000**	1.057(1.017–1.098)	**0.005**
Age			1.002(1.001–1.004)	**0.007**
Gender				
Male			1.0	
Female			1.097(1.070–1.126)	**0.000**
Ethnic group				
Han			1.0	
Ethnic minority			0.956(0.910–1.005)	0.077
Education				
Middle school or below			1.0	
High school or above			1.111(1.077–1.145)	**0.000**
Marital status				
Single ^a^			1.0	
Married ^b^			1.024(0.947–1.106)	0.552
Movement area				
Inter-provincial			1.0	
Intra-provincial			1.882(1.828–1.937)	**0.000**
Time in inflow area				
≤ 1			1.0	
> 1			1.206(1.170–1.242)	**0.000**
Plans for long-term residence (> 5 years)				
Yes			1.0	
No			0.646(0.618–0.674)	**0.000**
Not decided yet			0.833(0.808–0.858)	**0.000**
Number of children				
< 2			1.0	
≥2			0.917(0.892–0.943)	**0.000**
Employment status				
Employee			1.0	
Employer			1.143(1.091–1.197)	**0.000**
Self-supporting laborers			1.146(1.115–1.177)	**0.000**
Others			0.967(0.873–1.072)	0.523
Health insurance				
Yes			1.0	
No			0.784(0.742–0.829)	**0.000**
Family monthly income in the last year ^c^				
Q1			1.0	
Q2			0.969(0.938–1.001)	0.059
Q3			0.909(0.875–0.945)	**0.000**
Q4			0.849(0.815–0.884)	**0.000**
Constant	0.533	0.000	0.327	**0.000**
R squared		0.001		0.049
Observations	112,782			

^a^ Single includes those who are divorced (2.3%) and widowed (0.5%); The *P*-values in boldface mean statistical significance

^b^ Married includes those who are first married (95.5%) and remarried (1.7%)

^c^ Quartile 1 (Q1) is the poorest and Quartile 4 (Q4) is the richest

Univariate analysis indicates that migrants of rural origin are more likely to have established health records if they are female (*P* = 0.000), have education levels of high school or above (*P* = 0.000), are inter-province migrants (*P* = 0.000), have lived in their current residence for more than 1 year (*P* = 0.000), have work status of “employer” (*P* = 0.000), and are self-supporting laborers (*P* = 0.000). Migrants of rural origin are less likely to have established health records if they have no plans of long-term residence (*P* = 0.000) or haven’t decided yet (*P* = 0.000), have 2 or more children (*P* = 0.000), have no insurance (*P* = 0.000), and have higher family monthly income in the last 4 years (*P* = 0.000). As for migrants with urban origin, the univariate analysis shows that they are more likely to have established health records if they are older (*P* = 0.021), are inter-province migrants (*P* = 0.000), and are self-supporting laborers (*P* = 0.004). Migrants with urban origin are less likely to have established health records if they are ethnic minorities (*P* = 0.049), have education levels of high school or above (*P* = 0.026), have no plans for long-term residence (*P* = 0.000), have no insurance (*P* = 0.000), and are in the richest quartile (see Additional file 1: Table S1).

Table 3 presents the results of a multi-logistic analysis of factors associated with establishing health records in migrants with rural and urban origin. Factors found to be associated with the establishment of health records for migrants with rural origin during univariate analysis were all identified as determinants in the multivariate analysis as well. For migrants with urban origin, the multi-logistic regression indicates that migrants who are inter-province migrants (*P* = 0.000), and who are employers (*P* = 0.048) are more likely to have established health records. Those who are ethnic minorities (*P* = 0.002), who have no plans for long-term residence (*P* = 0.000), who have no insurance (*P* = 0.000), and who are in the richest quartile (*P* = 0.000) are less likely to have established health records.

**Table 3:** Multivariate analysis of factors associated with establishing health records in rural and urban migrant population in China, 2015

Characteristics	Rural migrants		Urban migrants	
	OR a (95%CI)	*P*	OR a (95%CI)	*P*
Age	NA		1.002(0.998–1.006)	0.357
Gender			NA	
Male	1.0			
Female	1.100(1.070–1.131)	**0.000**		
Ethnic group	NA			
Han			1.0	
Ethnic minority			0.797(0.692–0.917)	**0.002**
Education				
Middle school or below	1.0		1.0	
High school or above	1.128(1.092–1.165)	**0.000**	0.982(0.910–1.060)	0.641
Movement area				
Inter-provincial	1.0		1.0	
Intra-provincial	1.900(1.842–1.960)	**0.000**	1.710(1.585–1.846)	**0.000**
Time in inflow area			NA	
≤ 1	1.0			
> 1	1.243(1.204–1.284)	**0.000**		
Plans for long-term residence (> 5 years)				
Yes	1.0		1.0	
No	0.643(0.613–0.673)	**0.000**	0.663(0.589–0.746)	**0.000**
Not decided yet	0.809(0.783–0.836)	**0.000**	1.009(0.928–1.097)	0.829
Number of children			NA	
< 2	1.0			
≥ 2	0.922(0.896–0.948)	**0.000**		
Employment status				
Employee	1.0		1.0	
Employer	1.138(1.081–1.197)	**0.000**	1.119(1.001–1.251)	0.048
Self-supporting laborers	1.149(1.116–1.183)	**0.000**	1.076(0.994–1.165)	0.071
Others	0.971(0.863–1.093)	0.631	0.993(0.806–1.222)	0.944
Health insurance				
Yes	1.0		1.0	
No	0.783(0.735–0.834)	**0.000**	0.786(0.701–0.881)	**0.000**
Household monthly income in the last year ^a^				
Q1	1.0		1.0	
Q2	0.961(0.928–0.994)	**0.022**	1.025(0.932–1.126)	0.612
Q3	0.894(0.858–0.931)	**0.000**	1.000(0.901–1.111)	0.997
Q4	0.860(0.823–0.898)	**0.000**	0.830(0.749–0.920)	**0.000**

OR _a_: adjusted odds ratio; NA: Not applicable; The *P*-values in boldface mean statistical significance

^a^ Quartile 1 (Q1) is the poorest and Quartile 4 (Q4) is the richest

## Discussion

Our study finds that 35.1% of the migrant population has established health records in their local communities, which is higher than past research has found. A 2013 study showed that 16.9% of China’s migrant population had established health records [17]. Two 2014 studies in Sichuan province and Shanghai found that 25.2% and 18.9% of the migrant populations had established health records, respectively [18, 19]. In the past five years, the Chinese government has taken several measures to promote equal access to basic public health services among residents, including migrants, and progress resulting from these measures can already be seen. However, there is still a long way to go in this respect, and policymakers must continue to develop targeted interventions to improve the utilization of essential public health services among migrants.

The current study indicates that migrants of urban origin are more likely to have established health records than those of rural origin, which demonstrates that *Hukou* origin is associated with establishment of health records among migrants. This finding is supported by a study by Jiang et al., which also found that migrants with urban origin are more likely to have established health records than their counterparts of rural origin [20]. This can likely be explained by the fact that compared with migrants of urban origin, migrants of rural origin are more marginalized in many ways. One study shows strong evidence of marginalization experienced by migrant employees of rural origin, including lower employment and working conditions, lack of access to social security and medical benefits, difficulties in providing education to children, and barriers to housing [21]. In contrast, migrants of urban origin are more adaptive to local urban communities, which makes them more proactive about using public services, including medical services and health records.

We also found that intra-provincial migrant status, employer status, plans for long-term residence, possession of insurance, and lower household income are predictors for establishment of health records among migrants of both rural and urban origins. Intra-provincial migrants are also more likely to establish health records in their inflow communities. When migrants’ place of origin is closer to their new residence, lifestyle and health-related policy tend to be more similar between the two locations. Thus, these migrants are more able and willing to establish health records in their communities. We also find that migrants with work status as employers are more likely to establish health records. A study in the US finds that many employers provide sponsored personal health records for their employees, which indicates that employers might be more aware about the importance of establishing health records [22].

Migrants with no plans for long-term residence are also less likely to establish health records in local communities. Those who do not plan to reside long-term in their inflow areas may not have stable work and as a result may be less willing to establish health records. Migrants without insurance are also less likely to establish health records. Health records have been identified as an important tool for people to manage their own health, improve patient-physician communication [23], and facilitate continuity of care [24]. Those who have no health insurance might have low awareness of their own health and may not know the benefits of health records which could be the reason why they do not establish health records. Interestingly, higher household income is found to be negatively associated with establishment of health records among the migrants, which is inconsistent with previous studies [19]. Those migrants who have higher income might have more stressful work, and thus have less time to make use of public health services [25].

Among migrants of rural origin, we find that women are more likely to establish health records than men, which is consistent with previous studies [26]. We also find that those with education levels of high school or above tend to establish health records. This could be the effect of higher education on health consciousness or the indirect effect of education on employment characteristics. Migrants who have lived in their local area for more than one year are more likely to establish health records. The longer they stay in one place, the more familiar they are with that place. A longer stay also means stability which contributes to the establishment of health records. A study by Jiang et al. finds that the number of children is not associated with establishment of health records [20]. In contrast, our research shows that those who have two or more children are less likely to establish health records.

Among migrants of urban origin, ethnic minority migrants are less likely to establish health records than Han migrants. One possible explanation for this finding might be that the awareness of preventive health services is important in determining the establishment of health records in local communities. Some previous studies indicate that awareness of public health services is poorer among ethnic minority people than among Han people, and such awareness will inevitably affect whether they establish health records in inflow communities [27, 28]. Another explanation might be that the average education level of Hans is slightly higher than that of other ethnicities, which has been demonstrated in previous studies, and it is also true in this study. This may explain, to some extent, why ethnic minority urban migrants are less likely to establish health records. It is important to develop targeted interventions to promote basic public health services among ethnic minority migrants.

## Conclusion

Our study suggests that origin residence is associated with establishment of health records among migrant population in China, and specific interventions should be developed to improve essential public health services use among migrants of rural origin. This study also implies a need for more diverse health education for the migrant population. The government should also give more financial support to provide public health services such as continuous health records management for migrant populations. Furthermore, we identified some risk factors for establishment of health records among the migrant population of rural and urban origin separately. Policies should be designed to target the needs of these different subgroups.

## Additional file

Additional file 1: **Table S1.** Univariate analysis of factors associated with establishing health records in rural and urban migrant population in China, 2015. We presented univariate analysis of different factors associated with establishing health records in rural and urban migrant population respectively in this table. (DOCX 22 kb)
