# Supplementary material for: Does Hukou origin affect establishment of health records in migrant inflow communities? A nation-wide empirical study in China
Source: BMC Health Serv Res. 2018 Sep 10;18:704. doi: 10.1186/s12913-018-3519-6 (PMC6131757; doi:10.1186/s12913-018-3519-6)
Supplement: Supplementary file 1 — Table S1. Univariate analysis of factors associated with establishing health records in rural and urban migrant population in China, 2015. We presented univariate analysis of different factors associated with establishing health records in rural and urban migrant population respectively in this table. (DOCX 22 kb) [file 12913_2018_3519_MOESM1_ESM.docx]

Additional file 1: Table S1. Univariate analysis of factors associated with establishing health records in rural and urban migrant population in China, 2015

| **Characteristics** | **Rural migrant population** | | | | **Urban migrant population** | | | |
| --- | --- | --- | --- | --- | --- | --- | --- | --- |
|  | **Health records** | | **OR c (95%CI)** | ***P*** | **Health records** | | **OR c (95%CI)** | ***P*** |
|  | **Yes (%)** | **No (%)** |  |  | **Yes (%)** | **No (%)** |  |  |
| n | 33605(34.8) | 63045(65.2) |  |  | 6032(37.4) | 10100(62.6) |  |  |
| **Age** | 37.21(8.29) | 37.16(8.49) | 1.001(0.999-1.002) | 0.379 | 37.70(8.19) | 37.39(8.08) | 1.005(1.001-1.009) | 0.021 |
| **Gender** |  |  |  |  |  |  |  |  |
| Male | 19195(33.9) | 37390(66.1) | 1.0 |  | 3523(37.0) | 6002(63.0) | 1.0 |  |
| Female | 14410(36.0) | 25655(64.0) | 1.094(1.065-1.124) | **0.000** | 2509(38.0) | 4098(62.0) | 1.043(0.978-1.113) | 0.202 |
| **Ethic group** |  |  |  |  |  |  |  |  |
| Han | 31152(34.7) | 58650(65.3) | 1.0 |  | 5706(37.6) | 9478(62.4) | 1.0 |  |
| Ethnic minority | 2453(35.8) | 4395(64.2) | 1.051(0.998-1.106) | 0.058 | 326(34.4) | 622(65.6) | 0.871(0.758-0.999) | **0.049** |
| **Education** |  |  |  |  |  |  |  |  |
| Middle school or below | 24812(33.8) | 48575(66.2) | 1.0 |  | 1866(38.7) | 2957(61.3) | 1.0 |  |
| High school or above | 8793(37.8) | 14470(62.2) | 1.190(1.154-1.227) | **0.000** | 4166(36.8) | 7143(63.2) | 0.924(0.862-0.991) | **0.026** |
| **Marital status** |  |  |  |  |  |  |  |  |
| Single ^a^ | 818(34.6) | 1549(65.4) | 1.0 |  | 290(38.8) | 458(61.2) | 1.0 |  |
| Married ^b^ | 32787(34.8) | 61496(65.2) | 1.010(0.927-1.100) | 0.827 | 5742(37.3) | 9642(62.7) | 0.941(0.809-1.093) | 0.425 |
| **Movement area** |  |  |  |  |  |  |  |  |
| Inter-provincial | 13591(27.3) | 36284(72.7) | 1.0 |  | 2384(30.2) | 5509(69.8) | 1.0 |  |
| Intra-provincial | 20014(72.7) | 26761(27.3) | 2.021(1.960-2.084) | **0.000** | 3648(69.8) | 4591(30.2) | 1.780(1.653-1.917) | **0.000** |
| **Time in inflow area** |  |  |  |  |  |  |  |  |
| ≤1 | 7612(69.9) | 17696(30.1) | 1.0 |  | 1371(36.8) | 2359(63.2) | 1.0 |  |
| >1 | 25993(63.6) | 45349(36.4) | 1.332(1.292-1.374) | **0.000** | 4661(37.6) | 7741(62.4) | 1.036(0.960-1.118) | 0.360 |
| **Plans for long-term residence** (>5 years) | |  |  |  |  |  |  |  |
| Yes | 22690(38.0) | 37068(62.0) | 1.0 |  | 4371(38.1) | 7108(61.9) | 1.0 |  |
| No | 2885(25.7) | 8347(74.3) | 0.565(0.540-0.591) | **0.000** | 448(29.6) | 1068(70.4) | 0.682(0.607-0.766) | **0.000** |
| Not decided yet | 8030(31.3) | 17630(68.7) | 0.744(0.721-0.768) | **0.000** | 1213(38.7) | 1924(61.3) | 1.025(0.945-1.112) | 0.547 |
| **Number of children** |  |  |  |  |  |  |  |  |
| <2 | 19260(35.8) | 34503(64.2) | 1.0 |  | 4990(37.5) | 8310(62.5) | 1.0 |  |
| ≥2 | 14345(33.4) | 28542(66.6) | 0.900(0.877-0.925) | **0.000** | 1042(36.8) | 1790(63.2) | 0.969(0.891-1.054) | 0.469 |
| **Employment status** |  |  |  |  |  |  |  |  |
| Employee | 15386(32.7) | 31712(67.3) | 1.0 |  | 3666(36.5) | 6378(63.5) | 1.0 |  |
| Employer | 5237(63.8) | 2970(36.2) | 1.169(1.113-1.228) | **0.000** | 622(37.9) | 1021(62.1) | 1.060(0.952-1.180) | 0.290 |
| Self-supporting laborers | 25186(63.0) | 14814(37.0) | 1.212(1.179-1.247) | **0.000** | 1583(39.1) | 2463(60.9) | 1.118(1.037-1.205) | **0.004** |
| Others | 435(32.3) | 910(67.7) | 0.985(0.877-1.106) | 0.801 | 161(40.4) | 238(59.6) | 1.177(0.960-1.443) | 0.118 |
| **Health insurance** |  |  |  |  |  |  |  |  |
| Yes | 32152(35.2) | 59318(64.8) | 1.0 |  | 5521(37.8) | 9074(62.2) | 1.0 |  |
| No | 1453(28.1) | 3727(71.9) | 0.719(0.676-0.765) | **0.000** | 511(33.2) | 1026(66.8) | 0.819(0.732-0.915) | **0.000** |
| **Household monthly income in the last year** ^c^ | |  |  |  |  |  |  |  |
| Q1 | 9279(36.5) | 16160(63.5) | 1.0 |  | 1217(40.0) | 1829(60.0) | 1.0 |  |
| Q2 | 12546(35.3) | 22978(64.7) | 0.951(0.920-0.983) | **0.003** | 1956(40.1) | 2923(59.9) | 1.006(0.917-1.103) | 0.904 |
| Q3 | 6389(33.0) | 13000(67.0) | 0.856(0.823-0.890) | **0.000** | 1263(38.8) | 1989(61.2) | 0.954(0.863-1.056) | 0.365 |
| Q4 | 5391(33.1) | 10907(66.9) | 0.861(0.826-0.897) | **0.000** | 1596(32.2) | 3359(67.8) | 0.714(0.650-0.784) | **0.000** |

OR _c_: crude odds ratio

OR _a_ : adjusted odds ratio

Age is presented as Mean(Standare deviation).

^a^ Single includes those who are divorced and widowed.

^b^ Married includes those who are first married and remarried.

^c^ Quartile 1 (Q1) is the poorest and Quartile 4 (Q4) is the richest.
